# The International Deep Brain Stimulation Registry and Database for Gilles de la Tourette Syndrome: How Does It Work?

**DOI:** 10.3389/fnins.2016.00170

**Published:** 2016-04-25

**Authors:** Wissam Deeb, Peter J. Rossi, Mauro Porta, Veerle Visser-Vandewalle, Domenico Servello, Peter Silburn, Terry Coyne, James F. Leckman, Thomas Foltynie, Marwan Hariz, Eileen M. Joyce, Ludvic Zrinzo, Zinovia Kefalopoulou, Marie-Laure Welter, Carine Karachi, Luc Mallet, Jean-Luc Houeto, Joohi Shahed-Jimenez, Fan-Gang Meng, Bryan T. Klassen, Alon Y. Mogilner, Michael H. Pourfar, Jens Kuhn, L. Ackermans, Takanobu Kaido, Yasin Temel, Robert E. Gross, Harrison C. Walker, Andres M. Lozano, Suketu M. Khandhar, Benjamin L. Walter, Ellen Walter, Zoltan Mari, Barbara K. Changizi, Elena Moro, Juan C. Baldermann, Daniel Huys, S. Elizabeth Zauber, Lauren E. Schrock, Jian-Guo Zhang, Wei Hu, Kelly D. Foote, Kyle Rizer, Jonathan W. Mink, Douglas W. Woods, Aysegul Gunduz, Michael S. Okun

**Affiliations:** ^1^Department of Neurology, University of Florida and Center for Movement Disorders and NeurorestorationGainesville, FL, USA; ^2^Tourette's Syndrome and Movement Disorders Center, Galeazzi HospitalMilan, Italy; ^3^Department of Stereotactic and Functional Neurosurgery, University of CologneCologne, Germany; ^4^Neusurgical Department, IRCCS GaleazziMilan, Italy; ^5^Asia-Pacific Centre for Neuromodulation, Queensland Brain InstituteBrisbane, Queensland, Australia; ^6^University of Queensland Centre for Clinical Research, The University of QueenslandBrisbane, Queensland, Australia; ^7^BrizBrain&SpineBrisbane, QLD, Australia; ^8^Departments of Psychiatry, Pediatrics and Psychology, Child Study Center, Yale UniversityNew Haven, CT, USA; ^9^Sobell Department of Motor Neuroscience, University College London Institute of NeurologyLondon, UK; ^10^Assistance publique – Hôpitaux de Paris, Institut du Cerveau et de la Moelle Epiniere, Institut National de la Santé et de la Recherche Médicale 1127, Pitié-Salpêtrière Hospital, Sorbonne Universités, UPMC Univ Paris 06, Unité Mixte de Recherche 1127, Centre National de la Recherche Scientifique, Unité Mixte de Recherche 7225Paris, France; ^11^Institut National de la Santé et de la Recherche Médicale U 1127, Centre National de la Recherche Scientifique UMR 7225, Sorbonne Universités, UPMC Univ Paris 06 UMR S 1127, Institut du Cerveau et de la Moelle épinièreParis, France; ^12^Department of Neurosurgery, Assistance Publique – Hôpitaux de Paris, Hôpital de la Pitié-SalpêtrièreParis, France; ^13^Assistance publique – Hôpitaux de Paris, DHU Pe-PSY, Pôle de Psychiatrie et d'addictologie des Hôpitaux Universitaires H Mondor, Université Paris Est CréteilCréteil, France; ^14^Department of Mental Health and Psychiatry, Geneva University HospitalGeneva, Switzerland; ^15^Service de Neurologie, Institut National de la Santé et de la Recherche Médicale-Centres d'Investigation Clinique 1402, Centre Hospitalier Universitaire de Grenoble de Poitiers, Université de PoitiersPoitiers, France; ^16^Parkinson's Disease Center and Movement Disorders Clinic, Baylor College of MedicineHouston, TX, USA; ^17^Beijing Neurosurgical Institute, Capital Medical UniversityBeijing, China; ^18^Department of Neurology, Mayo Clinic College of MedicineRochester, MN, USA; ^19^Department of Neurosurgery, Center for Neuromodulation, NYU Langone Medical CenterNew York, NY, USA; ^20^Department of Psychiatry and Psychotherapy, University of CologneCologne, Germany; ^21^Department of Neurosurgery, Maastricht University Medical CentreMaastricht, Netherlands; ^22^Department of Neurosurgery, National Center Hospital, National Center of Neurology and PsychiatryKodaira, Japan; ^23^Department of Neurosurgery, Maastricht University Medical CenterMaastricht, Netherlands; ^24^Faculty of Health, Medicine and Life Sciences, School for Mental Health and Neuroscience, Maastricht UniversityMaastricht, Netherlands; ^25^Department of Neurosurgery, Emory UniversityAtlanta, GA, USA; ^26^Department of Neurology, Department of Biomedical Engineering, University of Alabama at BirminghamBirmingham, AL, USA; ^27^Division of Neurosurgery, University of TorontoToronto, Canada; ^28^Department of Neurology, The Permanente Medical Group (Tidewater Physicians Multispecialty Group), Movement Disorders ProgramSacramento, CA, USA; ^29^University Hospitals, Case Western Reserve University School of MedicineCleveland, OH, USA; ^30^Department of Neurology, University Hospitals Case Medical Center, Neurological InstituteCleveland, OH, USA; ^31^Parkinson's & Movement Disorder Center/Division, Johns Hopkins University, School of MedicineBaltimore, MD, USA; ^32^Department of Neurology, The Ohio State University Wexner Medical CenterColumbus, OH, USA; ^33^Division of Neurology, Centre Hospitalier Universitaire de Grenoble Grenoble, Grenoble Alpes UniversityGrenoble, France; ^34^Department of Psychiatry and Psychotherapy, Universitätsklinikum KölnKöln, Germany; ^35^Department of Neurology, Indiana University School of MedicineIndianapolis, IN, USA; ^36^Department of Neurology, University of UtahSalt Lake City, UT, USA; ^37^Department of Functional Neurosurgery, Beijing Tiantan Hospital, Capital Medical UniversityBeijing, China; ^38^Department of Neurological Surgery, University of FloridaGainesville, FL, USA; ^39^Department of Neurology, University of Rochester Medical CenterRochester, NY, USA; ^40^Department of Psychology, Marquette UniversityMilwaukee, WI, USA; ^41^J. Crayton Pruitt Family Department of Biomedical Engineering, University of FloridaGainesville, FL, USA

**Keywords:** Tourette syndrome, deep brain stimulation, database, registry, tics, regulatory agencies

## Abstract

Tourette Syndrome (TS) is a neuropsychiatric disease characterized by a combination of motor and vocal tics. Deep brain stimulation (DBS), already widely utilized for Parkinson's disease and other movement disorders, is an emerging therapy for select and severe cases of TS that are resistant to medication and behavioral therapy. Over the last two decades, DBS has been used experimentally to manage severe TS cases. The results of case reports and small case series have been variable but in general positive. The reported interventions have, however, been variable, and there remain non-standardized selection criteria, various brain targets, differences in hardware, as well as variability in the programming parameters utilized. DBS centers perform only a handful of TS DBS cases each year, making large-scale outcomes difficult to study and to interpret. These limitations, coupled with the variable effect of surgery, and the overall small numbers of TS patients with DBS worldwide, have delayed regulatory agency approval (e.g., FDA and equivalent agencies around the world). The Tourette Association of America, in response to the worldwide need for a more organized and collaborative effort, launched an international TS DBS registry and database. The main goal of the project has been to share data, uncover best practices, improve outcomes, and to provide critical information to regulatory agencies. The international registry and database has improved the communication and collaboration among TS DBS centers worldwide. In this paper we will review some of the key operation details for the international TS DBS database and registry.

## Introduction

Gilles de la Tourette Syndrome (TS) is a neuropsychiatric disorder characterized by motor and vocal tics. In a subset of cases, these tics can be severely debilitating (Freeman et al., 2000; Malaty and Akbar, 2014; Shprecher et al., 2014). The pathophysiology of TS has been increasingly linked to dysfunction in a complex basal ganglia thalamo-cortical circuit (BGTCC) (Da Cunha et al., 2015). Deep brain stimulation (DBS)—effective for movement disorders including Parkinson's disease, dystonia, and tremor—has been explored since 1999 as a potential therapy for select cases of severe, medication-resistant TS (Müller-Vahl et al., 2011). However, DBS use in TS is still considered investigational and has not received regulatory agency approval.

Initial stereotactic surgical treatment with thalamotomy for TS was introduced by Rolf Hassler in 1970 (Hassler and Dieckmann, 1970). Cooper, Hassler, and Dieckmann were part of surgical teams performing this procedure for few TS patients. Hassler initially targeted the centromedian-parafascicular complex. Thus, the selection of the thalamic target for DBS was motivated by the relative successes of Hassler and other clinicians applying the thalamotomy procedure in this brain region.

Despite the initial successes, thalamotomy was never widely adopted as a treatment for TS. The invasiveness of the procedure, the issues with accuracy using early stereotaxic equipment, and the risk of speech, swallowing, and cognitive side effects due to the large size of the lesions all limited its widespread use. Three decades later in 1999 Vandewalle and colleagues implanted DBS electrodes bilaterally in the nucleus ventro-oralis internus/centromedian-parafascicular complex (Voi/CM/Pf) of the thalamus (Vandewalle et al., 1999). The Vandewalle group was able to demonstrate the relative safety and potential effectiveness in a small series of patients published over the next several years. This initial experience sparked the interest of other groups and led to a dialogue about the possibility of applying DBS in various brain targets along the BGTCC.

This interest has been supported by a growing number of studies in the peer-reviewed literature (Ackermans et al., 2011; Massano et al., 2013; Jimenez-Shahed, 2015; Kefalopoulou et al., 2015). These studies reveal generally positive results with occasional side effects (e.g., hemorrhage, stimulation-induced), however it should be kept in mind that most studies have been small and uncontrolled (Duits et al., 2012; Sachdev et al., 2012; Savica et al., 2012; Ackermans et al., 2013; Dehning et al., 2014; Kim and Pouratian, 2014; Malaty and Akbar, 2014; Zhang et al., 2014; Kefalopoulou et al., 2015). Additionally, there were other important differences in the DBS intervention such as the brain target (Martínez-Fernández et al., 2011; Viswanathan et al., 2012), the surgical targeting methods, the type(s) of devices implanted, the stimulation paradigm (Rotsides and Mammis, 2013), and the baseline disease characteristics (Okun et al., 2008).

Teams performing DBS have in the past decade explored at least eight possible brain targets for TS cases (Cavanna et al., 2011; Porta et al., 2012). These targets have included the thalamic CM/Pf (Visser-Vandewalle et al., 2003; Maciunas et al., 2007; Ackermans et al., 2010, 2011), the subthalamic nucleus, the posterolateral globus pallidus internus, the anteromedial globus pallidus internus (Dehning et al., 2008; Massano et al., 2013; Dong et al., 2014), the globus pallidus externus (Piedimonte et al., 2013), the nucleus accumbens (Kuhn et al., 2007; Sachdev et al., 2012), the dorsomedial nucleus of the thalamus, and the anterior limb of the internal capsule (Flaherty et al., 2005).

Academic medical centers with specialized TS clinics have collectively reported only a handful of appropriate DBS candidates presenting for a surgical intervention each year, rendering it nearly impossible to achieve the statistical power necessary to draw critical conclusions about DBS therapy in this population. We therefore aimed to develop an International DBS Registry and Database for TS with the idea that the statistical power necessary to refine and improve this procedure could only be achieved through the collection of a large worldwide community of cases.

Questions to be answered include best targets, best phenotypical indications, most appropriate surgical and programming approaches, efficacy, and other outcomes. There are many obstacles for investigator initiated device studies as noted recently by Foote et al (Kelly et al., 2014). This is most problematic in less common disorders such as TS. Limited funding and lack of insurance coverage for devices in clinical trials have created a vicious cycle discouraging investigator-initiated device trials (Kelly et al., 2014; Rossi et al., 2014). The TS registry and database has the potential to facilitate a paradigm shift by collecting important information about TS DBS that cannot be obtained by using standard clinical trial design. One important goal of this project is to obtain approval for the procedure from appropriate regulatory agencies.

## Review of the literature

A review of the English language literature was performed through PUBMED using the medical subheading database with the keywords “deep brain stimulation” AND “Tourette syndrome.” The review was focused on original articles and excluded review articles.

A large number of reports were available, however most were case reports or small series. A relatively recent article by Motlagh et al. (2013) reviewed the available published cases. In Table 1, we summarize the studies reporting a minimum of four TS DBS patients. We excluded reports with less than four patients.

**Table 1:** **This table summarizes the published literature about DBS in Tourette Syndrome with number of subjects ≥ 4**.

**References**	***n***	**Age (years)**	**Gender**	**Target**	**Laterality of stimulation**	**Follow-up time**	**High frequency stimulation**	**Continuous stimulation**	**Tic improvement**	**Study country**	**Year published**	**Double blind randomized trial**
Servello et al., 2008	18	17–47	15 m, 3 f	Centromedian-parafascicular and ventralis oralis complex of the thalamus	Bilateral	3–18 months	Yes	Yes	YGTSS decreased from 33–48 to 7–22	Italy	2008	No
Motlagh et al., 2013	8	16–48	8 m, 0 f	Thalamus (5) and Globus pallidus internus (3—two in the sensorimotor portion and one in limbic portion)	Bilateral	6–107 months	Yes	Yes	YGTSS decreased by 0–72%	USA	2013	No
Maciunas et al., 2007	5	18–34	NA	Centromedian-parafascicular and ventralis oralis complex of the thalamus	Blinded off-off, off-on, on-off, on-on combinations of 1 week each, then open-label bilateral	3 months	Yes	Yes	three of five patients showed improvement, mean preop YGTSS 37.2, 3-month score 28.2	USA	2007	Yes (cross-over design)
Servello et al., 2009	4	25–47	3 m, 1 f	Internal capsule/nucleus accumbens in patients with centromedian-parafascicular and ventralis oralis complex of the thalamus (except one patient with only internal capsule/nucleus accumbens leads)	Bilateral	8–51 months	Yes	Yes	two patients showed at best mild improvement in OCD and tic scores, two showed more clinically significant improvement in OCD scores and functionality, with limited effect on tics	Italy	2009	No (case-series)
Porta et al., 2009	15	17–47	12 m, 3 f	Centromedian-parafascicular and ventralis oralis complex of the thalamus	Bilateral	24 months	Yes	Yes	Persistent improvement in tic scores. No deleterious effect on cognition, improvement in behavioral ratings	Italy	2009	No
Ackermans et al., 2010	6	28–42	6 m, 0 f	Centromedian-parafascicular and ventralis oralis complex of the thalamus	Bilateral, 3 months of either on or off, then 6 months on	12 months	Yes	Yes	YGTSS decreased from a mean of 42.3 prior to surgery to 21.5 on 1 year follow-up, *p* = 0.028	Netherlands	2010	Yes (cross-over design)
Martínez-Fernández et al., 2011	5	21–60	5 m, 0 f	Globus pallidus internus (two patients with anteromedial location, two patients with posterolateral location, one patient initially with posterolateral switched after 18 months to antermedial location	Bilateral	3–24 months	Yes	Yes	Mean YGTSS was 77.8 at baseline and 54.2 at last follow up, mean MRVRS was 28.3 at baseline and 15.7 at last follow up, TSQOL was 61.7 at baseline and 28.5 at last follow up	UK	2011	No (case-series)
Dehning et al., 2011	4	25–44	1 m, 3 f	Globus pallidus internus (posteroventrolateral location)	Bilateral	5–48 months	Yes	Yes	two patients responded with > 80% reduction in tics, two patients were non-responders	Germany	2011	No (case-series)
Cannon et al., 2012	11	18–50	8 m, 3 f	Globus pallidus internus (anteromedial location)	Bilateral	4–30 months	Yes	Yes	one patient was a non-responder; mean YGTSS was 84.45 before surgery and 42.55 at 3 months, mean TSQOL was 39.09 before surgery and 79.09 at 3 months	Australia	2012	No
Porta et al., 2012	18	17–47	15 m, 3 f	Centromedian-parafascicular and ventralis oralis complex of the thalamus	Bilateral	5–6 years	Yes	Yes	Mean YGTSS was 80.83 prior to surgery and 22.11 at the extended follow up (*p* < 0.001)	Italy	2012	No
Maling et al., 2012	5	28–39	2 m, 3 f	Centromedian-parafascicular and ventralis oralis complex of the thalamus	Bilateral	6 months	Yes	Yes	YGTSS decreased by 1–41%; noted correlation between gamma band activity change and YGTSS change after DBS	USA	2012	No
Okun et al., 2013	5	28–39	2 m, 3 f	Centromedian-parafascicular and ventralis oralis complex of the thalamus	Bilateral	6 months	Yes	No	YGTSS decreased by 17.8 points (*p* = 0.01), MRVRS decreased by 5.8 points (*p* = 0.01)	USA	2013	No
Dehning et al., 2014	6	19–39	3 m, 3 f	Globus pallidus internus (posteroventrolateral location)	Bilateral	12–60 months	Yes	Yes	two patients were non-responders, mean YGTSS was 90.2 prior to surgery and 29.5 at last follow up (*p* = 0.001), TSQOL was 88.75 prior to surgery and 7.75 at last follow up (one person did not fill TSQOL)	Germany	2013	No
Zhang et al., 2014	13	16–34	12 m, 1 f	Globus pallidus internus (posterolateral location)	Bilateral	13–80 months	Yes	Yes	Mean YGTSS decreased by 52.1% at last follow up, mean TSQOL improved by 45.7% at last follow up	China	2014	No
Huys et al., 2014	8	19–56	5 m, 3 f	Ventral anterior and ventrolateral motor parts of the thalamus	Bilateral except in two patients unilateral	12 months	Yes	Yes	YGTSS motor, impairment and total scores decreased by 51, 60, and 58% respectively compared to baseline. MRVRS score decreased by 58%. Significant improvement in quality of life and global functioning measures were noted	Germany	2014	No
Kefalopoulou et al., 2015	15	24–55	11 m, 4 f	Globus pallidus internus (anteromedial location)	Bilateral, 3 months on or off, then open label on stimulation	6 months blinded and then 8–36 months unblinded	Yes	Yes	YGTSS decreased by 12.4 between on and off states in the blinded phase (*p* = 0.048), YGTSS decreased by 23.8–48.9 points (*p* < 0.0001) between baseline and open label phase	UK	2015	Yes (cross-over design)

The most recent TS DBS study appeared in the Lancet Neurology in June 2015 (Kefalopoulou et al., 2015). It was a randomized double-blind crossover trial conducted in 15 patients. The target for most patients was the anteromedial GPi (two were targeted in the posteroventral GPi) and all subjects were randomly assigned 1:1 to either 3 months of on-stimulation or 3 months off stimulation. All subjects switched to the alternative condition. Only 13 of 15 patients completed the two-blinded assessments. There was a small benefit in tic reduction as noted by a mean improvement of 12.4 points (equivalent to 15.3%) on the Yale Global Tic Severity Scale (YGTSS).

Three other randomized double-blind trials have also been published. The target was CM/Pf and these studies had smaller numbers. Maciunas et al. (2007) randomized five patients to receive bilateral DBS electrode implantation in a single operative session. There was a standardized follow-up at 17–21 days following implantation. The first outcome was measured at 7 days, and patients were randomized to stimulation in one of four combinations (right on, left off; right off, left on; right on, left on; right off, left off). Each 7 days during a 28-day follow-up period another randomized outcome was implemented until all potential conditions were tested. This study procedure was then followed by 3 months of open label DBS. Tics were assessed by standardized rating scales and also by independent video analysis. Unilateral stimulation proved not as effective as bilateral DBS, and overall there were positive benefits in tic reduction reported in three of the five patients.

Ackermans et al. (2011) randomized six patients to receive bilateral DBS electrodes in the Voi/CM/Pf complex of the thalamus. Patients were assigned to 3 months on stimulation followed by 3 months off stimulation (group A) or vice-versa (group B). This crossover period was followed by 6 months of open label on stimulation. Only one patient was randomized to group B. There was a significant improvement of 37% in tics when comparing on vs. off states as well as at comparing baseline to final outcome. Assessments were performed using the Yale Global Tic Severity Scale. The authors noted that at 1 year, patients required more time to finish a selective attention and response inhibition test (Stroop Color Word Card Test).

Okun et al. (2013) randomized five patients who received bilateral DBS electrodes in the centromedian complex of the thalamus. A scheduled stimulation paradigm was used instead of the conventional continuous stimulation paradigm. Two patients were randomized to start stimulation at 30 days from implantation and the remaining three patients to start stimulation at 60 days from implantation. There was a statistically significant improvement in YGTSS total score (by 19%) and in the modified Rush Tic Rating Scale Score. The authors reported that tic suppression was most effective at deep contacts on the lead.

All of the studies published reported limitations and concerns regarding individual variability in outcome, the level of stimulation required, the effect of tolerance, battery life, electrical current spread, small sample size, and difficulty in maintaining the patient blinding.

## The international TS DBS registry and database: Goals and design

**Figure 1:**
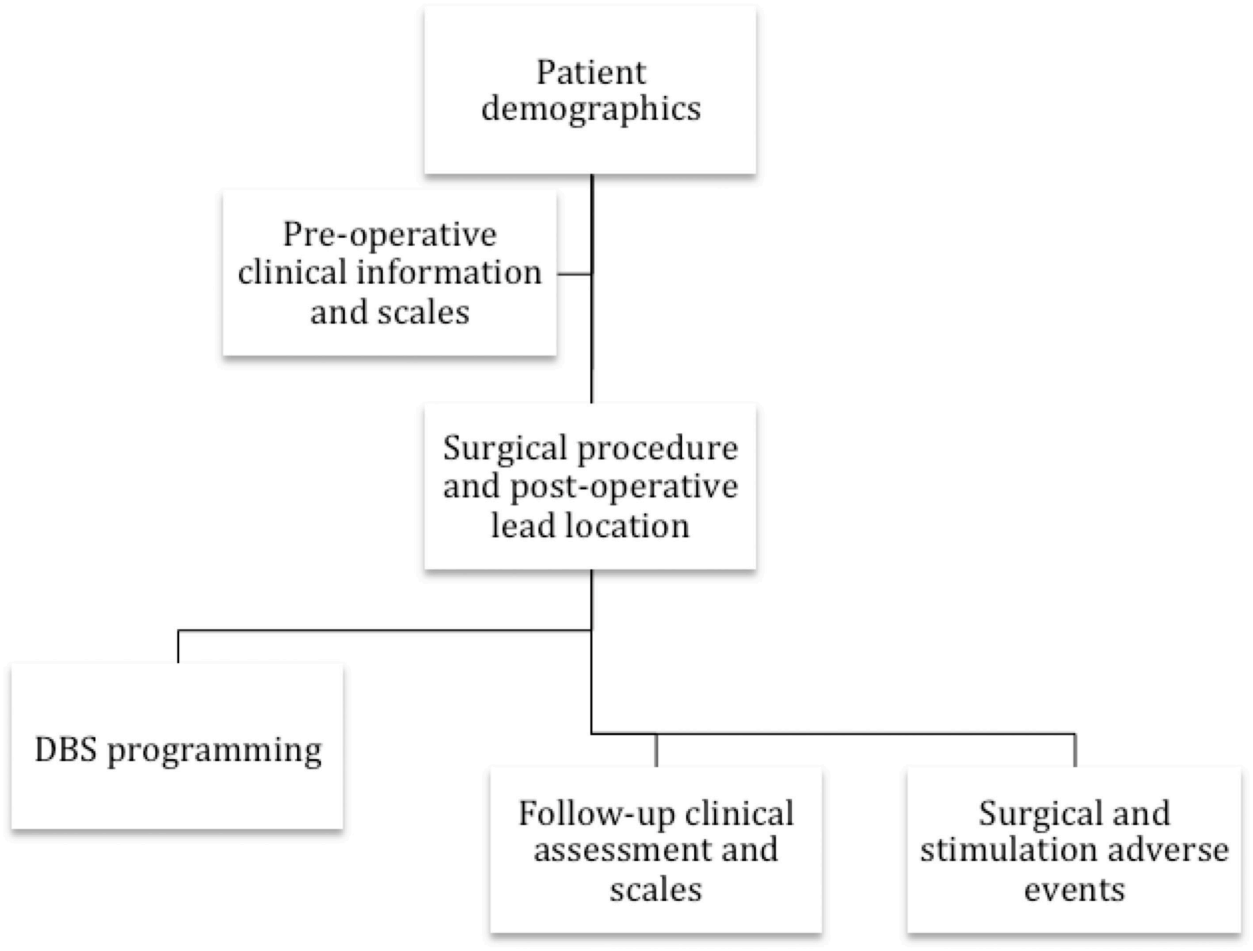
**The flowchart reveals the collaborative group's information collected on each TS DBS case**.

The international community collectively responded to the critical need in the DBS field by collaborating with the Tourette Syndrome Association (since renamed to the Tourette Association of America TAA) in 2012 and by launching an International TS DBS registry and database. The project sought to consolidate all of the information available for TS DBS cases worldwide. This effort aimed to shift the field from small case series and reports to an international large-scale collaborative effort. The elements included in the database are summarized in Figure 1.

This multi-center effort has resulted in the formation of a registry and a database. There is no restriction on investigators or groups wishing to join the project, and there is no limitation to the maximum data necessary to register a case. However, in order for the case to qualify for database status and outcome measurement, there must be a minimal amount of information available to facilitate a group analysis of all of the participating centers. Additionally, groups with negative as well as positive experiences with DBS cases are strongly encouraged to participate. Enrolling all subjects regardless of the quality of the outcome is mandatory and is an important factor to better understand the current state of the field.

The database and registry have facilitated networking of clinician-researchers and have led to the generation of new hypotheses for both research and care. The database and registry will provide a repository of valuable information for patient advocacy groups (e.g., TAA), device manufacturers, as well as third party payers who are keenly focused on the potential benefits, burdens, risks, and harms of the therapy.

The registry and database were constructed to collect information on each case (refer to Figure 1). Currently supported data have been divided into six categories: (1) demographic information and disease characteristics, (2) pre-operative clinical scales (i.e., Yale-Brown obsessive compulsive scale (YBOCS), YGTSS, Hamilton depression scale, etc.), (3), surgical procedure data (including brain target, targeting procedure, lead location, device type, and imaging data), (4) DBS programming parameters, (5), regular follow-up clinical assessment and scales, and (6) surgical as well as stimulation-related adverse events. Electrophysiological data from the DBS procedure is not currently being collected; however, efforts are underway to enable for those interested and familiar with the techniques, intraoperative single cell recordings, and local field potential (LFP) data extracted from next generation devices capable of chronic LFP recordings. As these devices become more widely used, these data will become more available and, importantly, could add to the insights into the physiology of tic and the mechanisms underlying DBS-related improvements in tic behaviors.

## Current status of the registry

Participants involved in the database include investigators who have been implanting TS DBS patients with and without the intent to publish.

To date 157 patients are registered from 10 different countries. 126 of the patients (80%) are male. The targets used include the thalamus (92 cases), anteromedial and posteroventral GPi (61 cases), and the anterior limb of the internal capsule/nucleus accumbens (2 cases).

The following are the most commonly submitted data:

1. Demographic data
  - Patient identifier
  - Gender
  - Country
  - Age at onset
  - Age at surgery
  - Co-morbidities with a specific focus on obsessive compulsive disorder (OCD)
  - List of medications tried and at the time of surgery
2. Pre-operative clinical scales
  - YGTSS at baseline
    - Total score
    - Motor tic subcomponent (less commonly available)
    - Vocal tic subcomponent (less commonly available)
    - Impairment subcomponent (less commonly available)
  - YBOCS at baseline
3. Surgical procedure data
  - Lead location and target
  - Device used
4. DBS programming parameters (limited data available at this point)
5. Follow up clinical assessment and scales
  - YGTSS at 1 year
    - Total score
  - YBOCS at 1 year
6. Adverse events

Another important objective of the database is to track safety. Many outcomes are collected and these outcomes have been aligned to the variables potentially necessary for a future humanitarian device exemption approval by regulatory agencies in different countries and regions. An adverse event form is available to participants in the database and was modeled after requirements from the American Food and Drug Administration (FDA). Participants have been asked to report all adverse events. The patient identifier has been used to link the adverse event to the patient. The following information has been collected: start date of adverse event, end date of adverse event, weight of patient at onset of event, outcome of adverse event (resolution, disability, hospitalization, death), description of the adverse event and associated relevant history, needed workup and laboratory studies, DBS hardware information (device name, serial number, implant date, explant date), and any therapies/surgeries needed as a result of the adverse event.

## Unique challenges facing the TS registry and database

There are many challenges facing an ambitious initiative, particularly of this size. One major challenge will be to assure data quality, particularly given the large number of participating centers. This challenge has been addressed by process refinement and feedback of data to the participating centers and sites. Frequent meetings of participating centers have been a critical element to improving data quality and also for informing sites about the minimum data necessary to move from a registration status to a full database status (submitting data about their own DBS in TS cases). Additionally, the database has fully dedicated support for data collection that is headquartered at the University of Florida Center for Movement Disorders and Neurorestoration. The central data repository has a full-time principal investigator (Professor Michael Okun) and a database manager who together are focused on the mission and objectives, defining policies and procedures, and assigning responsibilities for each participant. Additionally, the coordinating center has defined a clear communication plan, compliance monitoring, and data policy enforcement.

A more substantial challenge facing this international DBS registry will be to achieve data uniformity. Several scales exist to measure tics and each has advantages and limitations. Scales may assess one or more disease features (i.e., motor tics, vocal tics, OCD symptoms, and quality of life). There has been variability among groups in preferences for outcome measures and in time frames for assessment; standardization of submitted measures and clinical scales would allow more cases for analysis.

Another important issue facing TS DBS will be to ensure the database is highly accessible to its contributors and to promote transparency among investigators. This process if executed properly has the potential to instill confidence in contributors and to encourage programs to invest the resources necessary to obtain the critical measurements necessary for the success of the project.

## Strategies to achieve success

Several measures have been implemented to counteract potential database-related problems. One of the cornerstones of success will be continuous education of the investigators on data collection. The database has been purposely designed to draw in as many TS centers in the world participating in DBS operations. This large-scale effort will increase the number of patients and expand the potential for multiple data points for later analysis. Additionally, as centers enroll more patients the hope is that they will adapt and begin to collect more appropriate and relevant data-points.

An important strategy is scheduling regular meetings of the collaborating centers to foster cooperation and to provide updates on their progress and the obstacles faced. In June 2015, the second annual meeting was convened to discuss the Tourette database effort and was held at the World Congress on Tourette Syndrome and Tic Disorders (London, UK). Most TSA DBS contributors were in attendance and there were presenters from each country. An image registration initiative was launched to identify DBS lead locations within the cohort. The hope was that this initiative would substantially add to the lead localization images analyzed in conjunction with already collected information about DBS lead coordinates and programming parameters. This data may aid in the identification of the volumes of tissue activation across the targets and would facilitate the correlation to outcome. Another initiative was to locate a health economist to determine what information would be meaningful to collect across centers. One example of an immediate use of the data was a question raised at the annual investigator meeting. The group sought to answer whether there was an outcome difference between earlier vs. later DBS implantation. This type of collaborative meetings will be an important cornerstone for an international database, and the meetings will continue to create improvement opportunities and to answer new questions facing the field.

## TS registry and database role in regulatory agency approval processes

Another important goal of the database will be to facilitate applications to appropriate regulatory agencies worldwide for approval of TS DBS. This includes regulatory agencies worldwide such as FDA (USA), CE (European Union), PMDS (Japan), SFDA (China), TGA (Australia), and many other national and regional regulatory bodies. In the USA, the most likely approval would be through a FDA humanitarian device exemption given the small number of patients currently requiring therapy. DBS approval on a humanitarian basis for obsessive compulsive disorder was obtained using pooled data from several small n studies. The use of a database for TS will facilitate an analysis of a larger number of patients. It will facilitate the collection of important safety data, a crucial step needed for regulatory agency approval. The multi-center data collection will encourage a shift to a more uniform data collection and analysis.

## Conclusions

The international registry and database has been designed to overcome the severe limitations of *small-n* studies for TS DBS. The project has made considerable progress toward a truly global database. We have now demonstrated proof of principle that reliable and comprehensive data can be collected. This data will be used to address fundamental questions facing the TS DBS field, including identification of optimal brain target(s) for each patient based on individual symptom profiles, as well as stimulation parameters for each brain target and/or symptom. Additionally, a robust dataset will facilitate analysis of important questions that may potentially inform outcomes such as the relationship between baseline disease characteristics and the short and long-term clinical outcome. As data expand we will be able to move toward more advanced queries that can be used to address complex questions such as the relationship between electrode placement and clinical outcomes, as well as the correlation of lead location to adverse events. These basic, yet critical questions remain unanswered (Rotsides and Mammis, 2013; Jimenez-Shahed, 2015). Importantly, the systematic conglomeration of TS DBS datasets will generate the “higher n” critical to design clinical trials, power meaningful analyses, and generate recommendations for patient, target, and stimulation parameter selection. Finally, the database will be instrumental in applying for regulatory device exemptions.

## Author contributions

AM, AL, AG, BC, BW, BK, CK, DH, DS, DW, EJ, EM, EW, FM, HW, JL, JK, JZ, JH, JM, JS, JB, KF, LS, LA, LM, LZ, MW, MH, MP, MHP, PS, RG, SZ, SK, TK, TC, TF, VV, WH, YT, ZK, and ZM fulfilled the authorship criteria by substantial contributions to the conception of the work, revisiting it critically for important intellectual content, approving the final version, and agreeing to be accountable for all aspects of the work in ensuring that questions related to the accuracy or integrity of any part of the work are appropriately investigated and resolved. WD, PR, KR, and MO fulfilled the authorship criteria by substantial contributions to the design of the work and the acquisition, analysis, and interpretation of data for the work, drafting the work and revising it critically for important intellectual content, approving the final version to be published and agreeing to be accountable for all aspects of the work in ensuring that questions related to the accuracy or integrity of any part of the work are appropriately investigated and resolved.

## Funding

Tourette Association of America, National Institute of Health (NIH), R01 NR014852, and The NIH award supported MO's research.

### Conflict of interest statement

AM: Medtronic - consultant, Alpha-Omega engineering - consultant, honorarium; BC: received consultation fees for Medtronic; BW: paid consultant for Medtronic; DW: book royalties from Oxford University Press, Guilford Press, and Springer Press; speaking honoraria from the Tourette Syndrome Association; EJ: honoraria for speaking at meetings sponsored by Medtronic and St Jude Medical; EM: received honoraria for consulting services and lecturing from Medtronic; HW: receives support for research from the Bachmann Strauss Dystonia and Parkinson's Foundation and Medtronic; JL: received support from the National Institutes of Health (salary and research funding), Tourette Association of America (formerly the Tourette Syndrome Association), Grifols (formerly Talecris), UBS Optimus Foundation, the Open Road Alliance, John Wiley &amp; Sons (book royalties), McGraw-Hill (book royalties), and Oxford University Press (book royalties); JM: Consultant for Medtronic, Biomarin and Grant support from Abeona; JS: Consulting for medtronic and st jude medical; KF: Research and fellowship support from Medtronic. Research support from St. Jude, Boston Scientific, NeuroPace, and Functional Neuromodulation. No personal remuneration from industry sources; MH: honoraria for speaking at meetings sponsored by Medtronic and St Jude Medical; MHP: Dr. Pourfar receives consulting fees from Medtronic, Inc.; MO: research was supported by NIH R01 NR14852 and by the National Parkinson Foundation Center of Excellence at the University of Florida; PS: Consultant neurologist; RG: Medtronic, St. Jude Medical, Neuropace, MRI Interventions, Neuralstem, Sanbio; TC: Teaching honoraria from Medtronic &amp; Boston Scientific; TF: Dr Foltynie holds research grants from the Michael J Fox Foundation, Brain Research Trust, European Union FP7 scheme and the John Black Charitable Foundation. He has served on Data monitoring committees for Oxford Biomedica. He has received honoraria for speaking at meetings sponsored by Medtronic, St Jude Medical, UCB, Brittania Pharmaceuticals and Abbvie. VV: Personal Fees: Medtronic, St Jude Medical; Grants from Medtronic, St Jude medical, SAPIENS and Boston Scientific; Non-financial support from Medtronic, St Jude Medical, SAPIENS and Boston Scientific. The other authors declare that the research was conducted in the absence of any commercial or financial relationships that could be construed as a potential conflict of interest.
